# Baseline Skin Microbiota of the Leatherback Sea Turtle

**DOI:** 10.3390/microorganisms12050925

**Published:** 2024-05-01

**Authors:** Samantha G. Kuschke, Jeanette Wyneken, Debra Miller

**Affiliations:** 1 Department of Biomedical and Diagnostic Services, College of Veterinary Medicine, University of Tennessee, Knoxville, TN 37996, USA; skuschke@vols.utk.edu; 2 Department of Biological Sciences, Florida Atlantic University, Boca Raton, FL 33431, USA; jwyneken@fau.edu; 3 Center for Wildlife Health, University of Tennessee, Knoxville, TN 37996, USA; 4 One Health Initiative, University of Tennessee, Knoxville, TN 37996, USA; 5 Upwell, Monterey, CA 93940, USA

**Keywords:** leatherback sea turtle, skin microbiota, neonates, nesting females, next-generation sequencing

## Abstract

The integumentary system of the leatherback sea turtle (*Dermochelys coriacea*) is the most visible and defining difference of the species, with its smooth and waxy carapace and finely scaled skin, distinguishing it from the other six sea turtle species. The skin is the body’s largest organ and serves as a primary defense against the outside world and is thus essential to health. To date, we have begun to understand that the microorganisms located on the skin aid in these functions. However, many host–microbial interactions are not yet fully defined or understood. Prior to uncovering these crucial host–microbial interactions, we must first understand the communities of microorganisms present and how they differ through life-stage classes and across the body. Here, we present a comprehensive bacterial microbial profile on the skin of leatherbacks. Using next-generation sequencing (NGS), we identified the major groups of bacteria on the skin of neonates at emergence, neonates at 3–4 weeks of age (i.e., post-hatchlings), and nesting females. These data show that the predominant bacteria on the skin of the leatherback are different at each life-stage class sampled. This suggests that there is a shift in the microbial communities of the skin associated with life-stage class or even possibly age. We also found that different sample locations on the nesting female (i.e., carapace and front appendages = flipper) have significantly different communities of bacteria present. This is likely due to differences in the microhabitats of these anatomic locations and future studies should explore if this variation also holds true for neonates. These data define baseline skin microbiota on the leatherback and can serve as a foundation for additional work to broaden our understanding of the leatherbacks’ host–microbial interactions, the impacts of environmental changes or stressors over time, and even the pathogenicity of disease processes.

## 1. Introduction

The leatherback sea turtle (*Dermochelys coriacea*) is the largest species of sea turtle and the sole extant genus of the family Dermochelyidae [1]. One notable difference from other marine turtles is the leatherback integumentary system. In contrast to scalation and the heavy cornification of “hard-shelled” sea turtles, adult leatherback integument is pliable and waxy [1]. Neonatal leatherback skin is uniquely composed of small domed scales [1]. Leatherback integument is not only grossly different from that of other marine turtles but is also different in composition. The outermost layer of skin (stratum corneum) is composed mainly of resilient and flexible alpha-keratin underlying a thin layer of (tough) beta-keratin [1]. Other marine turtle species’ stratum corneum is composed of a thick layer of beta-keratin [1].

The integumentary system in leatherbacks plays functional roles in thermoregulation, osmotic balance, the prevention of dehydration, camouflage, the synthesis of vitamin D precursors, and as a barrier to infection and harmful wavelengths of light [1]. The skin is also host to a complex and dynamic community of microorganisms that together make up the skin microbiota. In recent years, the skin microbiota has been a major focus of research in humans and animals [2,3,4,5,6,7]. This surge in research stems from the growing understanding that microbiota, both skin and gut, can significantly impact host functions such as development, behavior, and health [8,9,10,11,12]. More specifically, skin microbiota are the primary defenses against invading pathogens and are thought to play significant roles in host health, immune function, host resistance, and responses to endogenous and exogenous stressors [9,10,11,12,13,14,15,16].

Research exploring the skin microbiota of marine animals has become an area of great interest because of the skin’s constant contact with seawater and the microorganism that live within it [17,18,19,20,21]. Investigations of marine mammals have begun to identify microbes that make up species-specific core microbiota [18,19,20,21]. Unfortunately, few studies have investigated skin microbiota in reptiles [5,10,22,23,24,25]. Moreover, the information on the bacterial microbiota of free-ranging sea turtles is limited, and even fewer integumentary flora studies have utilized contemporary culture-independent methods [6,10,25]. The reason for this data gap is likely multifactorial. In the case of leatherbacks, it is likely due to the unique challenges they pose to researchers both because of their pelagic oceanic lifestyle and the difficulty of maintaining them (at any life-stage class) in human care for research or rehabilitation [26,27]. However, investigations into the skin microbiota of leatherbacks can provide essential insight for conservationists, researchers, and rehabbers on their health [6]. Additionally, application of these data will broaden our understanding of the leatherbacks’ host–microbial interactions, the impacts of environmental changes or stressors over time, and even the pathogenicity of disease processes [6,10,28].

Prior to exploring host–microbial interactions, microbial baselines must be obtained. A robust survey of baseline skin microbiota in clinically healthy animals is necessary before its connection to health, disease, and environmental variables can be explored. Once baselines are obtained, research into the skin microbiota’s role in health, immune function, resistance to endogenous stressors, and disease can commence. In this study, we used next-generation sequencing (NGS) to identify and characterize the skin microbiota of leatherback sea turtles at different life-stage classes (neonates and nesting females).

## 2. Materials and Methods

### 2.1. Neonate Selection and Husbandry

The leatherback sea turtle neonates involved in this study originated from naturally oviposited nests on Juno Beach and Boca Raton, Florida. Neonates were collected during the 2021, 2022, and 2023 hatching seasons (May–August). Throughout each season, nests were routinely monitored for emergence. Upon emergence, all neonates were transported to the Florida Atlantic University (FAU) Marine Laboratory in Boca Raton, Florida, for assessment and entrance into the research colony. All neonates were visually examined within 12 h of emergence. Up to 5 neonates from each nest were selected for entrance into the research colony at the FAU Marine Laboratory. All of those selected for entrance into the colony appeared clinically normal (i.e., active, alert, and had no obvious external abnormalities). All neonates were housed indoors at the FAU Marine Laboratory. Atlantic ocean water was pumped in through a near shore collection and was filtered through two (100 μm) filtration socks, cleaned via protein skimming, and treated with ultraviolet light sufficient to eliminate microbes [28]. Water was also sent through a chiller to maintain appropriate temperatures for leatherback sea turtles (23–25 °C) [28]. Fluorescent lights (UVA-UVB) on a 12:12 h cycle were hung 45 cm above the tanks [28].

### 2.2. Neonate Sample Collection

Skin swabs were collected from neonates entering the FAU research colony on the day of emergence and again at 3–4 weeks of age. Sample collection followed the same procedure at both time points. Each neonate was rinsed with 50 mL of sterile nano-pure water four times. Due to their small size, a single sterile swab (rayon or polyester) was used to sample the animal’s whole integument, including the head, carapace, plastron, and all four limbs. Swabbing was repeated in duplicate for each neonate. Each swab was placed into a sterile vial and was stored at −80 °C until DNA extraction.

### 2.3. Nesting Female Selection and Sample Collection

All nesting females were sampled from Juno and Jupiter Beach, Florida. Samples were collected during the 2022 and 2023 nesting seasons. For each nesting female, sample collection began after initiation of egg deposition and the nesting fixed action patter, during which the nesting female is nonresponsive to manipulation [29]. Prior to sampling, a ~30 cm area on the cranial half of the carapace was rinsed with 500 mL of sterile nano-pure twice. Then, using sterile swabs (rayon or polyester) a ~10 cm by 10 cm area in the rinsed region was swabbed twice. The rinsing and swabbing process was then repeated on the proximal region of the flipper (left or right as available due to positioning). Samples were placed into individual sterile tubes and then on ice until they could be stored in a freezer at −80 °C until DNA extractions.

### 2.4. DNA Extractions

All DNA extractions were performed using the DNeasy^®^ PowerSoil^®^ Pro Kit (QIAGEN^®^, Hilden, Germany) according to manufacturer’s protocol with the following modifications. Swabs were placed into the PowerBead Pro Tubes with 800 μL of CD1 solution and left to sit for 5 min. PowerBead Pro Tubes were then vortexed for 10 min with the swabs still in the tubes. The supernatant was removed from the PowerBead Pro Tube from around the swab (i.e., the swab was still within the tube). The manufacturer’s protocol was then followed until the final step, which had the following modification. Solution C6 was left to sit on the center of the white filter membrane for 5 min prior to centrifuging. All DNA was stored at −80 °C until sequencing.

### 2.5. Next-Generation Sequencing

All DNA was submitted to the University of Tennessee Genomics Core for polymerase chain reaction (PCR) and NGS. Next-generation sequencing was performed using the V3-V4 region of the 16S rRNA gene with forward primer 5′ CCTACGGGNGGCWGCAG 3′ and reverse 5′ GACTACHVGGGTATCTAATCC 3′ on the Illumina MiSeq (San Diego, CA, USA) [30].

### 2.6. Data Processing

Sequence data were analyzed using the DADA2 pipeline v1. 18.0 [31,32] in RStudio v4.3.1 [33]. Taxonomy was assigned using the IDTAXA algorithm in the DECIPHER v3.18 package [34,35]. Samples with <10,000 reads were removed prior to statistical analysis.

### 2.7. Statistical Analysis

All data analyses were performed using Rstudio v4.3.1 [33]. Packages used included ‘phyloseq’ v1.41.1, ‘vegan’ v2.6-4, ‘devtools’ v2.4.5, ‘microbiome’ v3.18, and ‘ggplot2’ v30.3 [36,37,38,39,40]. Alpha diversity was explored using Observed OTUs, Shannon index, Chao1 estimates, and Inverse Simpson index. Alpha diversity comparisons between age classes and between sample location on nesting females were made using pairwise Wilcoxon rank sum tests with Holm *p*-values that were adjusted for multiple comparisons [41]. Beta diversity was explored using principal coordinate analysis (PCoA) (Bray–Curtis) and pairwise Adonis tests were used to compare between life-stage classes and between sample location on nesting females [41].

## 3. Results

### 3.1. Comparisons across Life-Stage Classes

**Figure 1:**
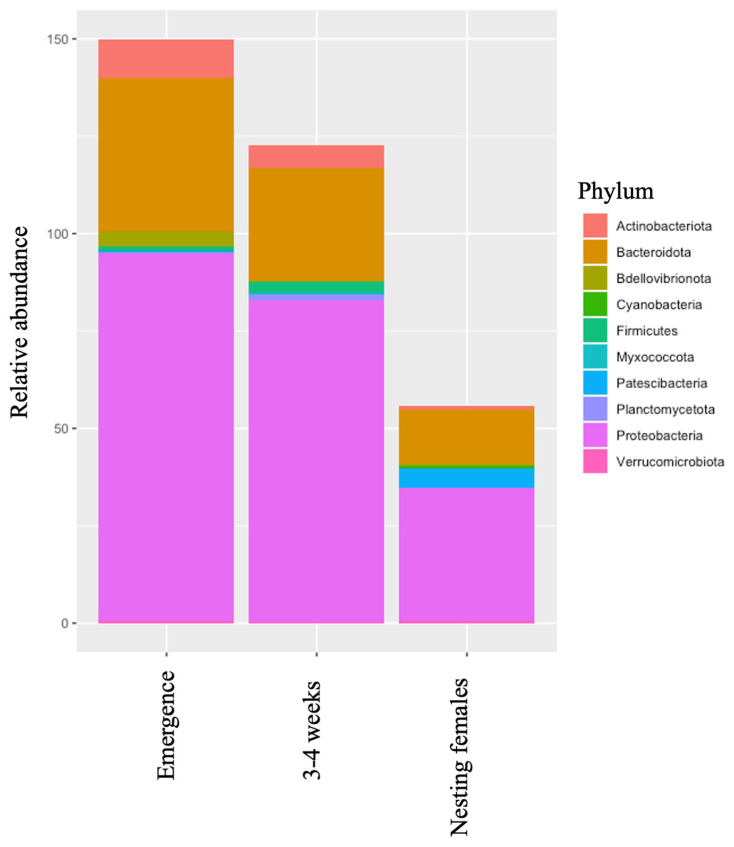
Relative abundance of top 10 bacterial phyla in different life-stage classes of leatherback sea turtles. Note that at the phylum level there are few differences between the life-stage classes. However, Patescibacteria are more prominent on the nesting females when compared to neonates at emergence or 3–4 weeks of age.

**Figure 2:**
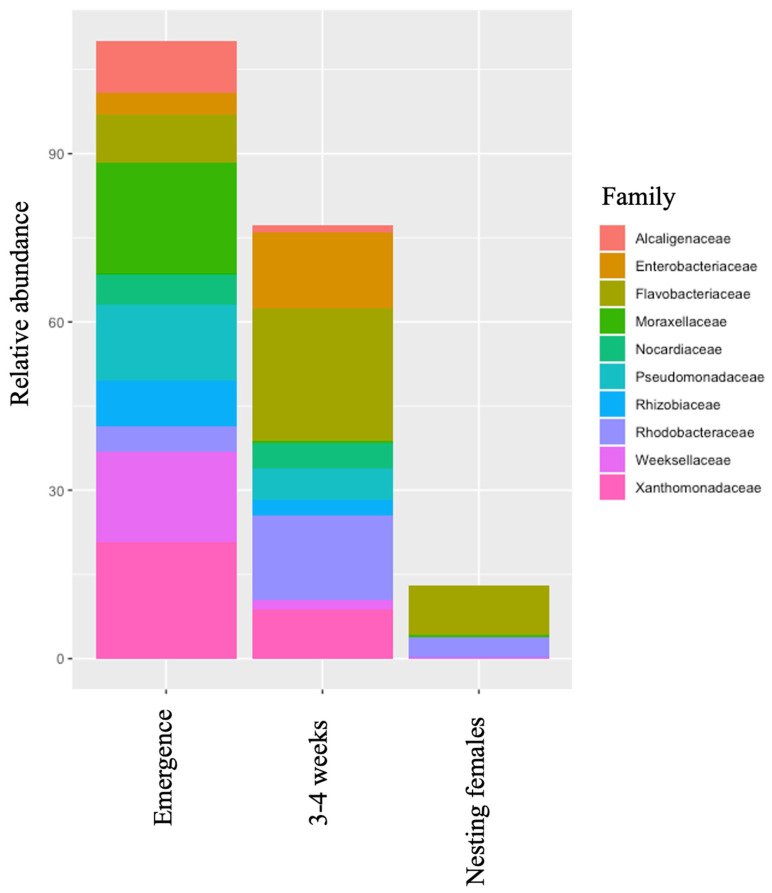
Relative abundance of top 10 bacterial families in different life-stage classes of leatherback sea turtles. A broad range of bacterial families was found on neonates at emergence with the most abundant families being Xanthomonadaceae and Moraxellaceae. In neonates at 3–4 weeks, the most abundant families shifted to Flavobacteriaceae and Rodobacteraceae. Flavobacteriaceae and Rodobacteraceae were also the most abundant bacterial families found on the nesting females.

From 2021 to 2023, a total of 178 neonatal leatherbacks from 38 nests were sampled at emergence and 140 of those turtles were also sampled again at 3–4 weeks of age (Table 1). During the 2022 and 2023 nesting seasons, a total of 37 nesting females were sampled (Table 1). Across all life-stage classes, five phyla were predominant: Proteobateria, Bacteroidota, Patescibacteria, Bdellovibrionota, and Firmicutes (Table 2, Figure 1). Relative abundance of each phylum was similar across all leatherbacks except for Patescibacteria, which was found in higher abundance in nesting females than in neonates at emergence or neonates at 3–4 weeks of age. There were six prominent families across all leatherbacks that included Flavobacteriaceae, Rodobacteraceae, Xanthomonadaceae, Moraxellaceae, Saprospiraceae, and Pseudomonaadaceae (Table 3, Figure 2). At emergence, the most abundant families were Xanthomonadaceae and Moraxellaceae. However, at 3–4 weeks, a shift in the most abundant families to Flavobacteriaceae and Rodobacteraceae was observed. Flavobacteriaceae and Rodobacteraceae were also the most abundant bacterial families found on the nesting females.

**Figure 3:**
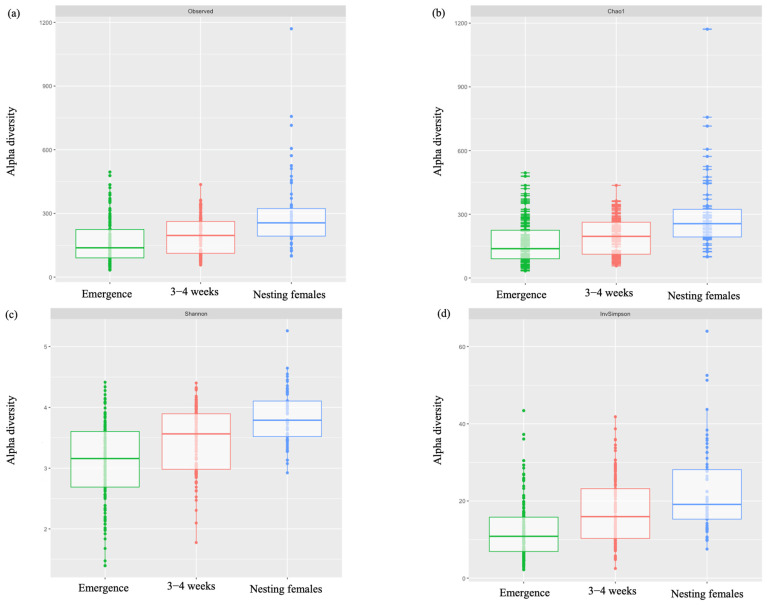
Alpha diversity across life-stage classes of leatherback sea turtles measured by (**a**) Observed OTUs, (**b**) Chao1 estimates, (**c**) Shannon, and (**d**) Inverse Simpson indices. Pairwise Wilcoxon rank sum tests with Holm *p*-values adjusted for multiple comparisons revealed significant differences among all groups for all measures of alpha diversity (df = 2). See Table 4 for *p*-values.

**Figure 4:**
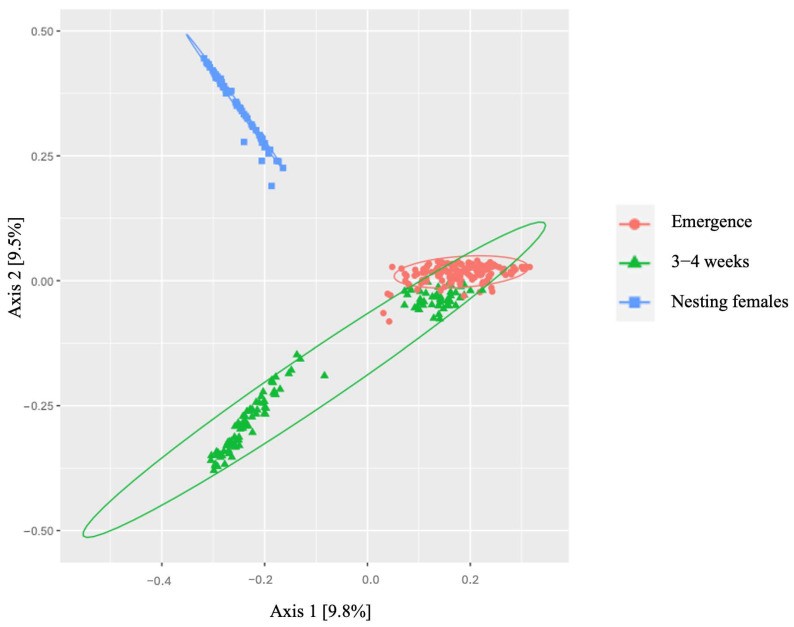
Beta diversity expressed using a principal coordinate analysis (PCoA) of Bray–Curtis distance showing differences in microbiota across all life-stage classes of leatherback sea turtles. This may be due to significant differences in microbial communities that exist between all life-stage classes. There is some overlap between neonates at emergence and neonates at 3–4 weeks. This may be due to a slower shift in the microbial community in some individuals. However, significant differences were still seen between all life-stage classes regardless of the overlap in neonates. See Table 5 for *p*-values.

Analysis of alpha diversity revealed significant differences between all life-stage classes in Observed Operational Taxonomic Units (OTUs) (df = 2, Figure 3a; Table 4) and in species richness between samples as measured by Chao1 estimates (df = 2 Figure 3b; Table 4), Shannon (df = 2, Figure 3c; Table 4), and Inverse Simpson indices (df = 2, Figure 3d; Table 4). For beta diversity, we detected clustering patterns for all life-stage classes of leatherbacks sampled (i.e., emergence, 3–4 weeks, and nesting females). Significant differences in microbial communities existed between all three life-stage classes (Figure 4; Table 5).

### 3.2. Comparisons between Carapace and Flipper on Nesting Females

**Figure 5:**
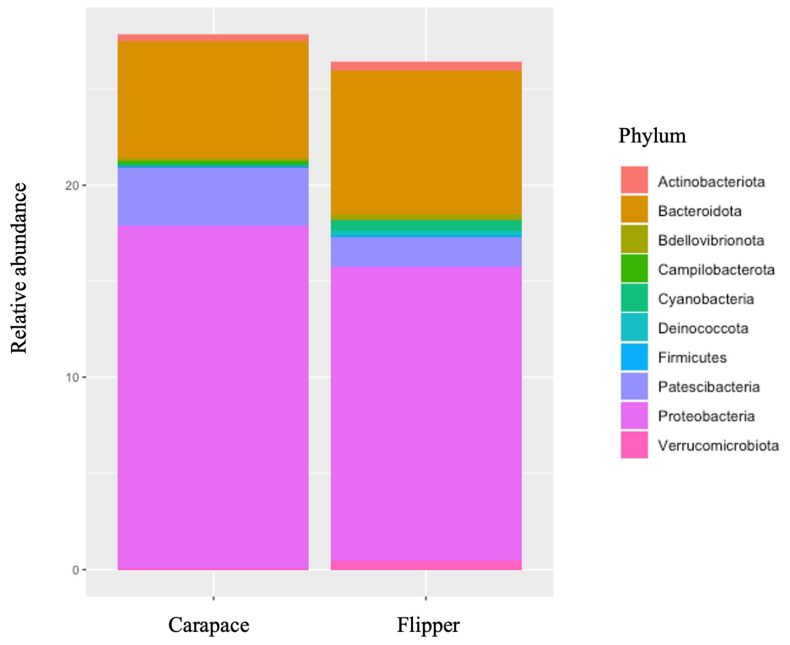
Relative abundance of bacterial phyla on the carapace and flipper of nesting leatherback sea turtles.

**Figure 6:**
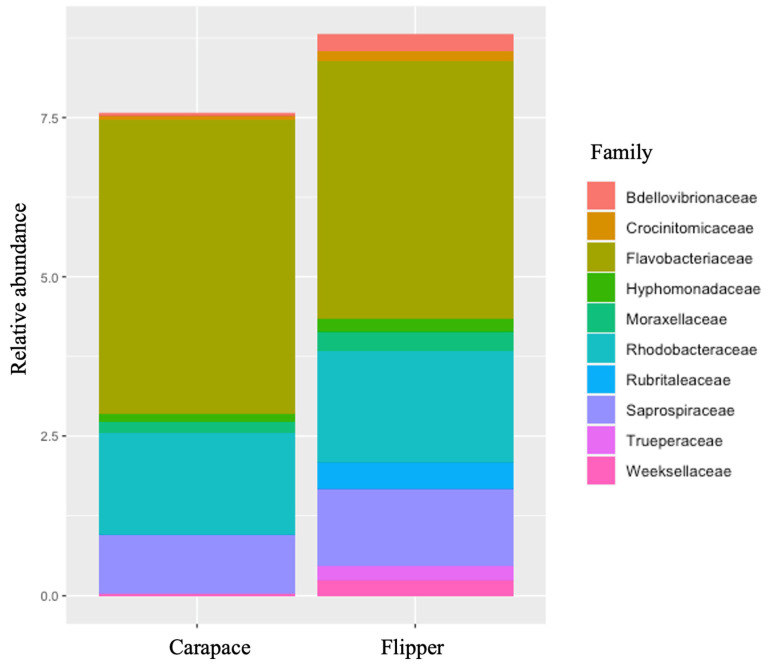
Relative abundance of bacterial families on the carapace and flipper of nesting leatherback sea turtles.

**Figure 7:**
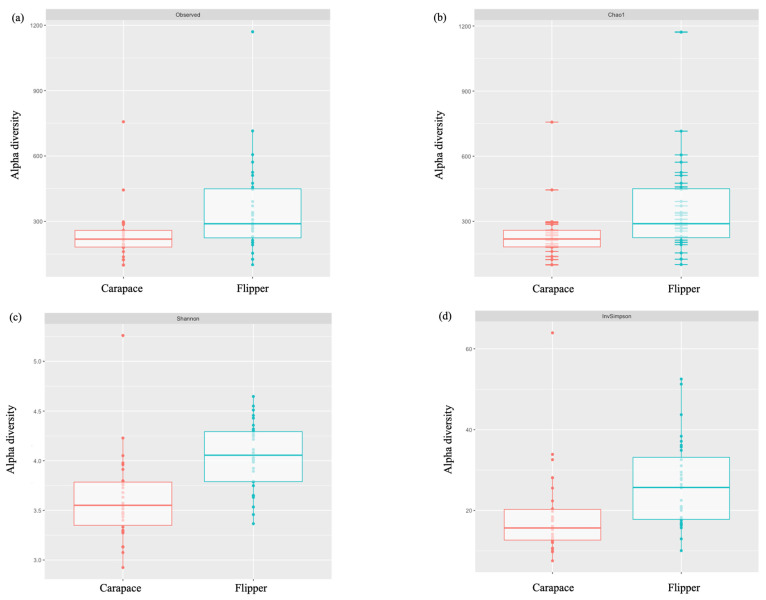
Alpha diversity of the carapace and flipper of nesting leatherbacks. Pairwise Wilcoxon rank sum tests with Holm *p*-values adjusted for multiple comparisons revealed significant differences in alpha diversity between sample location when measured by (**a**) Observed OTUs (df = 1, *p* = 0.004), (**b**) Chao1 estimates (df = 1, *p* = 0.004), (**c**) Shannon (df = 1, *p* < 0.001), and (**d**) Inverse Simpson indices (df = 1, *p* = 0.001).

**Figure 8:**
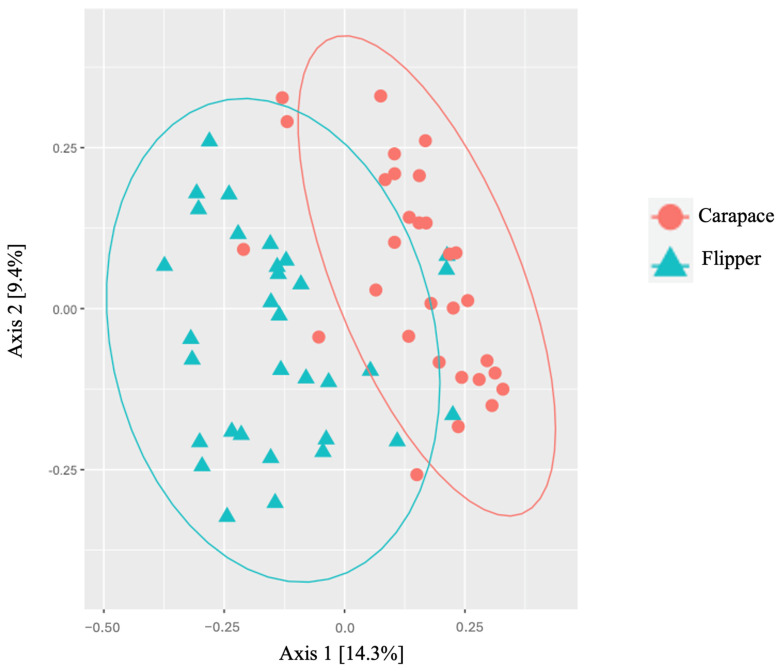
Beta diversity expressed using a principal coordinate analysis (PCoA) of Bray–Curtis distance showing differences in microbial diversity of the carapace and flipper on nesting leatherback sea turtles. Significant differences in microbial communities existed between locations (R^2^ = 0.09, *p* = 0.001).

Predominant phyla on both the carapace and flipper of nesting females were similar. However, Cyanobacteria and Verrucomicrobiota were both more predominant on the front flipper, while Patescibacteria was found in higher abundance on the carapace (Table 6, Figure 5). Bacterial families were also very similar between sample locations on the nesting females, except Rubritaleaceae was notably more abundant on the flipper (Table 7, Figure 6). Significant differences in alpha diversity were seen in Observed OTUs (df = 1, *p* = 0.004; Figure 7a) and in species richness between carapace and flipper samples as measured by Chao1 estimates (df = 1, *p* = 0.004; Figure 7b), Shannon (df = 1, *p* <0.001; Figure 7c), and Inverse Simpson indices (df = 1, *p* <0.001; Figure 7d). Analysis of beta diversity also revealed significant differences in microbial communities between the two sample locations (i.e., carapace and flipper) (R^2^ = 0.09, *p* = 0.001; Figure 8).

## 4. Discussion

In this study, we provide essential baseline microbiota data in leatherbacks and show that the composition of skin microbiota changes between life-stage classes and location on the body. These data fill a key knowledge gap for this species and can be used as a tool to assess population health and understand the impacts of environmental shifts [6]. Due to their long pelagic oceanic life history, sea turtles are often referred to as sentinel species [42,43,44]. As such, they are used to assess environmental changes and their potential impacts on marine life [42,43,44]. Due to the skin’s constant contact with seawater, it is an ideal location to assess the impacts of environmental factors on health [18,19,20,21]. Using these baseline data, researchers and conservationists can now begin to investigate host–microbial interactions, the impacts of environmental changes or stressors over time, and even extrapolate the pathogenicity of skin disease [6,10,28].

### 4.1. Comparisons across Life-Stage Classes

Across all life-stage classes, relative abundance was similar for the top two most predominant phyla (i.e., Proteobacteria and Bacteroidota). These results are similar to those reported in Mediterranean loggerheads (*Caretta caretta*) [25]. However, the third most predominant bacterial phylum in leatherbacks differs from that reported in loggerheads (i.e., Bdellovibrionota). In neonates, at emergence and 3–4 weeks of age, the third most predominant bacterial phylum was Actinobacteriota, and in nesting females, it was Patescibacteria. Patescibacteria was found in higher abundance in nesting females when compared to neonates at emergence (27× and 3–4 weeks (81×). This phylum was found in higher abundance in nesting females. The phylum Patescibacteria is considered a superphylum composed of a diverse set of bacteria [45,46]. Bacteria within this phylum are often found in aquatic environments [46,47,48,49]. Thus, it is likely that the abundance of this bacterial phylum on the skin increases with prolonged exposure to an aquatic environment (i.e., seawater) and therefore is found in higher abundance on the skin of nesting females. At the family level, there was a shift in the two most abundant families from Xanthomonadaceae and Moraxellaceae at emergence to Flavobacteriaceae and Rodobacteraceae at 3–4 weeks of age. This shift most likely reflects a normal life-stage class-related change in skin microbiota and is also likely related to the environmental shift the neonates make from the nest environment to the ocean. Both life stage and local environment are known to influence microbial communities [4,50,51,52]. These theories are further supported as the most prominent bacterial families on nesting females are also Flavobacteriacea and Rodobacteraceae.

Observations in alpha diversity reveal significant differences in the microbial diversity on individuals at each life-stage class. All measures of alpha diversity (i.e., Observed, Chao1, Shannon, and Inverse Simpson) reveal a trend of increasing alpha diversity over time. Additionally, the significant differences observed in beta diversity suggest that the communities present at each life-stage class are different. This suggests that the microbial communities present on the skin change throughout the life-stage classes measured here. Limited studies in reptiles make comparisons difficult; however, studies in humans have reported changes in skin microbiota related to age [50,53,54,55].

It is important to note that the leatherback neonates at 3–4 weeks of age included in this study were maintained in a laboratory environment. The ocean water the neonates are housed in is filtered and treated with UV light to kill any microbes present. Additionally, for biosecurity reasons, all neonates were handled minimally and only while using clean examination gloves. Consequently, the maintenance of these animals in this ‘clean’ environment likely has some impact on the skin microbiota. It has also been shown that captivity can impact the composition of microbiota [56,57,58]. Consequently, the microbiota data presented here for leatherback neonates at 3–4 weeks of age may not directly reflect their wild counterparts. However, due to limitations with accessing wild populations of neonatal leatherbacks, this is the closest approximation to healthy wild leatherback neonates currently available. In the future, if access allows, additional studies should be conducted on wild leatherback neonates. Though access to wild leatherback neonates is limited, a potential alternative next step to consider could be rearing leatherbacks in untreated and constantly circulating ocean water.

### 4.2. Comparisons between Carapace and Flipper on Nesting Females

The relative abundance of both bacterial phyla and families were relatively similar between the carapace and the flipper of nesting females. However, the phyla Cyanobacteria and Verrucomicrobiota were both more predominant on the flipper while Patescibacteria was found in higher abundance on the carapace. The bacterial family Rubritaleaceae was notably more abundant on the flipper. Alpha diversity was consistently higher on the flipper when compared to the carapace, and differences in beta diversity between locations show that the bacterial communities at each location were significantly different. Minor differences in relative abundance of phyla and families were expected, as were the significant differences in both alpha and beta diversity.

Previous studies in loggerheads and freshwater turtles have demonstrated significant differences in microbial communities on different parts of the body such as the head, skin, and shell [23,24,25]. Kanjer et al. (2022) reported higher diversity and richness on the carapace of loggerheads when compared to samples taken from the skin (i.e., head, neck, and flippers) [25]. This is likely due to microhabitats on the skin that influence the microbiota at different locations on the body [58]. In contrast to the loggerheads, I found significantly higher alpha diversity on the flippers of leatherbacks when compared to the carapace. This difference is likely due to the unique composition of the leatherbacks integumentary system. The adult leatherback carapace is a waxy, pliable surface composed of a resilient and flexible alpha-keratin underlying a thin layer of beta-keratin [1]. In contrast to this, the front flippers are covered in smooth and fine scalation [1]. The texture and compositional differences between these two regions likely create two unique microhabitats that may be more or less favorable to particular bacteria. The fine scalation on the front flipper likely allows the proliferation of a more diverse array of bacteria, resulting in the higher alpha diversity observed, though not significantly higher. The proteins forming the skin and scutes vary across the turtle bodies, contributing to the nature of microhabitats. Morphological work with several freshwater species document structural and biochemical diversity and nature of integument with body region [59,60].

Health assessments of nesting sea turtles are the most common due to the ease of accessing them during oviposition [61,62,63,64]. However, it is important to note that the physiologic state of the nesting female is highly unique and may influence the skin microbiota [65,66,67]. Leatherback sea turtles are capital breeders that forage little to none during nesting season [63,68]. In addition to fasting, a nesting female also undergoes changes in hormone levels associated with reproduction [65,66,67], which has been shown to impact skin microbiota in humans [66,69]. Consequently, the microbiota data presented here for nesting leatherbacks may not reflect the normal microbiota of a foraging leatherback [6]. As such, additional studies should be aimed at characterizing the normal microbiota of foraging leatherbacks, both male and female.

The differences between sample locations seen in nesting females may also be true for neonatal leatherbacks. In this study, the whole body of neonatal leatherbacks was swabbed due to their small size and the generally conserved fine scalation across their body [1]. However, given the observations in nesting females, differences between sample locations (i.e., carapace and limbs) should also be explored over time in neonates. As leatherback neonates grow and shed their scales, it is likely that differences in microbial communities will arise across the body and resemble that of the nesting female. The shift of these communities over time could then be used to make inferences to the role of the bacteria at different locations and during different life stages.

## 5. Conclusions

This study presents novel information on leatherback skin microbiota and reveals that a unique skin microbiota exists at different life-stage classes. Additionally, we found that different locations on the body host unique microbial communities. Moving forward, additional research should be aimed at identifying the core microbiota present on the skin or foraging leatherbacks and in wild neonates, if logistically feasible. However, these baseline data serve as an essential foundation to begin to understand the role that the skin microbiota plays in leatherback health, immune function, disease, and resilience to environmental stressors. Understanding these essential host–microbial relationships is the next step in managing this highly endangered species.

## Figures and Tables

**Table 1:** Summary of sequencing results.

	Turtles Sampled	Number of Samples Submitted for PCR and NGS	Samples Used for Analysis	Total Reads	Average Reads
Emergence	178	172	151	7,503,179	49,690
3–4 weeks	140	135	126	7,337,226	58,232
Nesting females	37	74 *	63	4,767,105	74,486

* This number includes swabs from both the flipper and carapace.

**Table 2:** Relative abundance of top 10 most abundant bacterial phyla across life-stage classes.

Phylum	Neonates at Emergence	Neonates at 3–4 Weeks	Nesting Females
Proteobacteria	63.0	67.5	60.6
Bacteroidota	26.2	23.8	25.3
Actinobacteriota	6.7	4.5	1.6
Patescibacteria	0.3	0.1	8.1
Bdellovibrionota	2.5	0.3	0.8
Firmicutes	0.8	2.2	0.2
Cyanobacteria	0.01	0.03	1.4
Planctomycetota	0.06	1.1	0.16
Verrucomicrobiota	0.39	0.08	0.97
Myxococcota	0.06	0.18	0.02

**Table 3:** Relative abundance of top 10 most abundant bacterial families across life-stage classes.

Family	Neonates at Emergence	Neonates at 3–4 Weeks	Nesting Females
Flavobacteriacea	5.7	21.1	50.1
Rhodobacteraceae	3.0	13.3	17.9
Xanthomonadaceae	14.2	7.0	0.02
Moraxellaceae	13.3	0.3	2.1
Alcaligenaceae	6.3	0.95	0.002
Pseudomonaadaceae	9.1	4.6	0.04
Weeksellaceae	10.8	1.3	1.6
Enterobacteriaceae	2.6	10.8	.0008
Rhizobiaceae	5.5	2.4	.04
Nocardiaceae	3.8	3.6	.004

**Table 4:** Pairwise Wilcoxon rank sum tests with Holm *p*-values adjusted for multiple comparisons across alpha diversity measures (i.e., Observed OTUs, Chao1, Shannon, and Inverse Simpson indices) revealed significant differences among all groups (df = 2).

	Emergence				3–4 Weeks			
Alpha Diversity Measure	Observed OTUs	Chao1	Shannon	Inverse Simpson	Observed OTUs	Chao1	Shannon	Inverse Simpson
**3–4 weeks**	0.003	0.003	<0.001	<0.001	--	--	--	--
**Nesting females**	<0.001	<0.001	<0.001	<0.001	<0.001	<0.001	<0.001	0.001

**Table 5:** *p*-values for the pairwise Adonis tests used to compare beta diversity between the life-stage classes.

	Emergence	3–4 Weeks
3–4 weeks	0.001	
Nesting females	0.001	0.001

**Table 6:** Relative abundance of top 10 bacterial phyla of different sample locations on nesting leatherback sea turtles.

Phylum	Carapace	Flipper
Proteobacteria	63.9	57.5
Bacteroidota	22.2	28.2
Patescibacteria	10.8	5.6
Actinobacteriota	1.3	1.8
Cyanobacteria	0.48	2.2
Verrucomicrobiota	0.09	1.8
Bdellovibrionota	0.49	1.2
Deinococcota	0.02	1.0
Campilobacterota	0.55	0.04
Firmicutes	0.09	0.25

**Table 7:** Relative abundance of the top 10 bacterial families of different sample locations of nesting leatherback sea turtles.

Family	Carapace	Flipper
Flavobacteriacea	58.9	41.8
Rodobacteraceae	17.5	18.3
Saprospiraceae	12.6	13.3
Moraxellaceae	1.4	2.9
Rubritaleaceae	0.04	3.9
Hyphomonadaceae	1.1	1.9
Bdellovibrionaceae	0.56	2.4
Weeksellaceae	0.22	2.8
Crocinitomicaceae	0.80	1.5
Trueperaceae	0.03	2.3

## Data Availability

The data presented in this study are available on request from the corresponding author due to ongoing use of the data for additional research aims. Upon completion of those aims, the data will be made available.
